# Symptomatic Pelvic Organ Prolapse and Self-Rated Health, One Year After Childbirth: A Swedish Nationwide Register Study

**DOI:** 10.1007/s00192-025-06322-8

**Published:** 2025-09-29

**Authors:** Maria Mirskaya, Anna Isaksson, Eva-Carin Lindgren, Ing-Marie Carlsson

**Affiliations:** Department of Health and Welfare, Halmstad University, SE-823, 301 18 Halmstad, Sweden

**Keywords:** Nationwide register study, One year after childbirth, Pelvic organ prolapse, Self-rated health, Symptomatic pelvic organ prolapse, Women of reproductive age

## Abstract

**Introduction and Hypothesis:**

Pelvic organ prolapse (POP) is a complication of childbirth that may impair the overall health of women. We hypothesized that women with symptomatic pelvic organ prolapse (sPOP) would rate their health lower than women without sPOP 1 year after childbirth.

**Methods:**

The Swedish National Pregnancy Register, and the Pregnancy Survey were merged and searched for women with data on self-rated health and POP 1 year after childbirth. The women (*n* = 43,082), who answered these validated questions in the Pregnancy Survey between December 2022 and October 2024 comprised our study population, of which 40,392 were included in the final analysis. Analysis was performed using descriptive statistics and a binary logistic regression model to estimate the associations between self-rated health and sPOP 1 year after childbirth.

**Results:**

In total, 5704 (13.2%) participants reported sPOP; 1617 (28.3%) women with sPOP and 6669 (17.8%) women without sPOP rated their health as poor. sPOP was associated with poor self-rated health 1 year after childbirth (OR 1.557, 95% CI 1.453–1.669). Additionally, the following covariates: low education, urinary incontinence, fecal incontinence, and poor self-rated health before pregnancy were also associated with poor self-rated health 1 year after childbirth.

**Conclusions:**

Women with sPOP had higher odds of reporting poor self-rated health 1 year after childbirth compared to women without sPOP. In Sweden, sPOP represents a public health concern affecting women in their prime years and may lead to poorer health outcomes throughout the rest of their lives.

## Introduction

Childbirth is often seen as a natural and welcome life event, typically accompanied by hopeful expectations. In high-income countries, it is assumed that women should not have to risk their health during or after bringing a child into the world. However, it is well known that women who give birth vaginally may suffer from pelvic floor dysfunction (PFD) [1], including pelvic organ prolapse (POP) [2–4]. Although symptomatic pelvic organ prolapse (sPOP) commonly manifests years after childbirth [4–6], it can also develop soon postpartum and persist due to severe damage to the pelvic floor’s supportive structures [3, 5].

Studies have shown that sPOP negatively affects women’s daily activities, relationships, and future family planning [7–10]. Moreover, sPOP has been linked to impaired sexual function, psychological health issues [11], and body image disturbance [12]. Approximately 7–10% of those who deliver vaginally will undergo POP surgery [3, 13].

Research on sPOP among younger, still-fertile women is scarce, and large-scale studies are lacking. This limits both clinical practice and the evidence base needed to underpin clinical guidelines and policy decisions. Self-rated health (SRH) is a widely used indicator that captures multiple dimensions of health and strongly correlates with specific health problems and morbidity [14–16]. However, research on SRH in relation to childbirth has largely overlooked pelvic floor dysfunctions (PFD), including sPOP [17, 18].

Therefore, the present study used nationwide data from the Swedish Pregnancy Register (SPR) to investigate the association between self-rated health and symptomatic pelvic organ prolapse 1 year after childbirth. We hypothesized that women with sPOP would rate their SRH lower than women without sPOP.

## Materials and Methods

### Study Design and Data Source

This register-based cohort study used data from the SPR and the Pregnancy Survey, which are Linked. The SPR is a national quality register that has been in its current form since 2014 [19]. The coverage rate for registered deliveries in 2023 was 98.4%. The SPR contains data prospectively on demographic, reproductive, and maternal health information, as well as details on childbirth and pregnancy outcomes for both the mother and the newborn. Beginning with the initial registration at the antenatal care and continuing through the postnatal follow-up, conducted 4–16 weeks postpartum [20]. Since December 2020, pregnant women and new mothers in Sweden have been offered the opportunity to complete the Pregnancy Survey. The Pregnancy Survey is sent out digitally to all pregnant women and new mothers whose data is registered in the SPR. It can be completed at three-time points: around 25 weeks of pregnancy (Pregnancy Survey 1), around 8 weeks after giving birth (Pregnancy Survey 2), and around 1 year after giving birth (Pregnancy Survey 3). The surveys include questions on patient-reported outcome measures (PROM), which measure patients’ views of their illnesses and health and patient-reported experience measures (PREM) focusing on the care process [20]. Pregnancy Survey 1 contains questions about SRH before pregnancy and experiences with care during pregnancy while Survey 2 addresses SRH and experiences with care during childbirth and early postpartum. Survey 3 covers mental and physical health postpartum, SRH, and care experiences 1 year after childbirth. Pregnancy Surveys 2 and 3 also include questions about urinary incontinence (UI), fecal incontinence (FI), and pelvic pain. Additionally, Survey 3 includes questions about sexual activity and sPOP. In summary, the PROM part of these surveys consists of a combination of various questions drawing from different validated measures with the addition of SRH [20]. To test the hypothesis posed in our study, data from Surveys 1 and 3 were selected.

Data from the SPR was delivered as anonymized data, and individual women could not be identified. Women receive written information about the SPR and Pregnancy Surveys during the first visit to the maternal health clinic and online; participation is voluntary, and they can opt out. Patients also have the right to demand that their personal information be erased from the SPR and surveys.

### Study Population

**Fig. 1:**
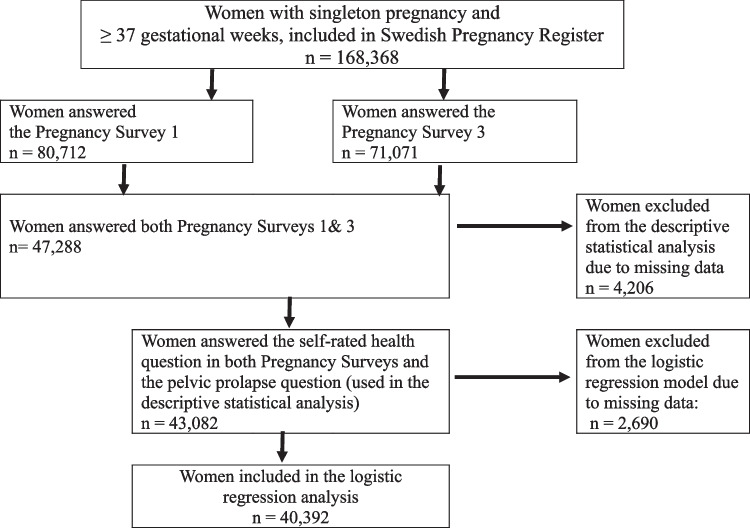
Flowchart of study population

In total, (*N* = 168,368) women who gave birth between January 2022 and October 2023 were retrieved from the SPR. Data were Linked to the Pregnancy Surveys 1 and 3. The response period for Pregnancy Survey 3, 1 year after birth, was between December 2022 and October 2024. The inclusion criteria were live term birth (≥ 37 gestational weeks); singleton pregnancy, and responses on SRH in both surveys, as well as on the question about POP. Of the 47,288 women (28%) who completed both surveys, 43,082 had complete SRH and POP data, resulting in a study population 43,082. A sensitivity analysis comparing nonresponders with the women in the study population showed that those included were slightly older (mean age 32.3 vs 31.6 years). Also, the results showed that a larger proportion of the women included in the study were first time mothers (49.3% vs. 41.2%). For the analytical statistics only participants who had provided responses to all questions were included (*n* = 40,392). For an illustration of the selection process see the flowchart in Fig. 1.

### Outcome Measures

SRH 1 year after giving birth was assessed in Pregnancy Survey 3, through the single question: “How would you rate your general state of health now?” Women responded using a 5-point Likert scale ranging from 1 (very poor) to 5 (very good), with labels provided only at the endpoints and no descriptors for points 2, 3, and 4. In line with previous research [18] these five response options were dichotomized into two groups: 1, 2, and 3 as poor SRH (coded as 1) and 4 and 5 as good SRH (coded as 0).

### Exposure Measures

sPOP was defined on the basis of a validated question in Pregnancy survey 3: “Do you have a sensation of tissue protrusion from the vagina?” [21]. Response options included: never, hardly ever, 1–3 times per month, 1–3 times per week, and daily. sPOP was classified as reporting symptoms of feeling vaginal bulge 1–3 times per month, 1–3 times per week, or daily in line with early research [2].

### Covariates and Sociodemographic Characteristics

SRH before pregnancy was collected from Pregnancy Survey 1, using the question: “How would you rate your general state of health before you got pregnant?”. It was dichotomized and coded in the same way as the outcome variable SRH, i.e., high value indicated poor SRH.

UI and FI data were extracted from Pregnancy Survey 3. UI was defined using the question: “Do you have involuntary loss of urine?” with response options: never, hardly ever, 1–3 times per month, 1–3 times per week, and daily. The responses were dichotomized into never or hardly ever versus 1–3 times per month, 1–3 times per week, or daily. A high value indicated the presence of symptomatic UI. FI was defined using the question: “Do you find it difficult to control your faeces or flatulence?” (categorized as the presence or absence of symptoms: yes/no where the high value indicated the presence of symptoms).

The sociodemographic characteristics of age, civil status, and parity were obtained from the SPR. The variable education (categorized as ≤ 12 years or > 12 years of schooling) was retrieved from the SPR, where a high value indicated lower education.

### Statistical Analyses

Statistical analyses were performed with SPSS (version 28, IBM Corp., Armonk, NY, USA). Descriptive statistics were used to describe sociodemographic and maternal characteristics and reported used numbers (*n*), proportions (%), medians, means and standard deviations (SD).

A binary logistic regression model, using enter method, was used to estimate the associations between SRH and sPOP 1 year after childbirth. In the model, education, pre-pregnancy SRH, UI, and FI were included as covariates. Odds ratios (OR) with 95% confidence intervals (CI) are presented with associated *p* values. A *p* value < 0.05 was considered to indicate a statistically significant association.

## Results

### Sample Characteristics

Sociodemographic maternal characteristics and characteristics in relation to SRH and PFD are shown in Table 1. All the women had a mean age of 32.3 ± 4.5 years when giving birth. For those who reported sPOP, the mean age was 32.6 ± 4.5, while for those without sPOP, it was 32.3 ± 4.5. Most of the women were cohabiting and had a university education. Almost half of the sample were primiparous with a mean age of 31.0 ± 4.4. Parity, expressed as mean and SD for the entire group, was 1.69 (0.852) with a median of 1. For the group with sPOP, the mean was 1.82 (0.897) with a median of 2, while for the group without sPOP, the mean was 1.67 (0.844) with a median of 1. The overall prevalence of sPOP was 13.2%.

**Table 1:** Sociodemographic characteristics and characteristics in relation to self-rated health and pelvic floor dysfunction in women with symptomatic pelvic organ prolapse and without symptomatic pelvic organ prolapse

		All women *n* = 43,082 (100%)	Women with sPOP *n* = 5704 (13.2%)	Women without sPOP *n* = 37,378 (86.8%)	*p* value* (chi-square**)
		*n* (%)	*n* (%)	*n* (%)	
Educational attainment					
	> 12 years of education	29,192 (71.6)	3917 (72.9)	25,275 (71.3)	** < 0.015**
	≤ 12 years of education	11,606 (28.4)	1453 (27.1)	10,153 (28.7)	Phi 0.012
Civil status					
	Cohabitant	38,306 (98.2)	5139 (98.1)	33,167 (98.3)	0.505
	Single	688 (1.8)	98 (1.9)	590 (1.7)	Phi 0.003
Parity					
	Primiparous	21,252 (49.3)	2401 (42.1)	18,851(50.4)	** < 0.001**
	Multiparous	21,830 (50.7)	3303 (57.9)	18,527 (49.6)	Phi 0.057
SRH before pregnancy					
	Good	38,659 (89.7)	4988 (87.4)	33,671 (90.1)	** < 0.001**
	Poor	4423 (10.3)	716 (12.6)	3707 (9.9)	Phi 0.029
SRH 1 year after childbirth					
	Good	34,796 (80.8)	4087 (71.7)	30,709 (82.2)	** < 0.001**
	Poor	8286 (19.2)	1617 (28.3)	6669 (17.8)	Phi 0.090
UI					
	No	31,787 (74)	3224 (56.8)	28,563 (76.7)	** < 0.001**
	Yes	11,155 (26)	2454 (43.2)	8701 (23.3)	Phi 0.153
FI					
	No	36,839 (86.2)	4196 (74.4)	32,643 (88)	** < 0.001**
	Yes	5879 (13.8)	1442 (25.6)	4437 (12)	Phi 0.134
Isolated sPOP		2565 (6)	2565 (45)		
Concomitant sPOP & UI		2454 (5.7)	2454 (43)		
Concomitant sPOP & FI		1142 (2.7)	1142 (20)		

Variables with missing data: educational attainment *n* = 2484 (5.3%), civil status *n* = 4088 (9.5%), UI *n* = 140 (0.3%), Concomitant sPOP & UI *n* = 140 (0.3%), FI *n* = 364 (0.8%), Concomitant sPOP & FI *n* = 364 (0.8%)

*sPOP* symptomatic pelvic prolapse, *SRH* self-rated health, *UI* urinary incontinence, *FI* fecal incontinence

^*^p < 0.05 indicates statistically significant relationship, ** comparison of women with and without sPOP

### Association Between Self-Rated Health and Symptomatic Pelvic Organ Prolapse One Year After Childbirth: A Logistic Regression Analysis

The logistic regression model was statistically significant, X2 (5, *n* = 40,392) = 2032.071 *p* < 0.001. In the model sPOP had a statistically significant association with SRH (OR 1.557, 95% CI 1.453–1.669). More specifically the reporting of sPOP was associated with poor SHR 1 year after childbirth. Also, the covariates: education, UI and FI, as well as SRH before pregnancy were associated with SRH 1 year after childbirth (for more information about estimates, see Table 2).

**Table 2:** Association between self-rated health (SHR) and symptomatic pelvic organ prolapse (sPOP) 1 year after childbirth adjusted for covariates (*n* = 40 392)^a,b^

Independent variables	B	*p value*	*OR*	*CI*
sPOP	0.443	< 0.001	1.557	1.453–1.669
Educational attainment	0.302	< 0.001	1.352	1.280–1.428
Self-rated health (SRH) before pregnancy	1.198	< 0.001	3.314	3.092–3.552
Urinary incontinence (UI)	0.425	< 0.001	1.529	1.445–1.617
Fecal incontinence (FI)	0.470	< 0.001	1.600	1.494–1.713

^a^For the regression analysis for sPOP, SRH, UI, and FI, values were recoded to ensure consistent directionality: higher values = more problems; for educational attainment: higher value = low education. Dependent variable SRH 1 year after childbirth (dichotomized 0 = good SHR; 1 = poor SHR)

^b^Independent variables in the analysis were sPOP (dichotomized 0 = no; 1 = yes), educational attainment (dichotomized 0 = > 12 years of education; 1 = ≤ 12 years of education), SRH before pregnancy (dichotomized 0 = good SHR; 1 = poor SHR), UI (dichotomized 0 = no; 1 = yes); FI (dichotomized 0 = no; 1 = yes)

*B* unstandardized regression coefficient, *OR* odd ratio, *CI* confidence interval

## Discussion

This study, based on nationwide register data from the SPR, examined the association between SRH and sPOP 1 year after childbirth. The results of this study confirm the hypothesis that women with sPOP rated their health lower than women without sPOP. Following adjustment for established sociodemographic risk factor (low education), SRH before pregnancy and other pelvic floor disorders, poor SRH remained associated with sPOP 1 year after childbirth.

The results align with previous studies on women of predominantly older ages, where the SRH item was included as part of other multidimensional measures such as the SF-12, SF-36, and the Pelvic Organ Prolapse Quality of Life questionnaire (P-QOL) [22–24]. In a larger review of studies on the biopsychosocial profile of women of various ages with sPOP, it was shown that sPOP had a moderate impact on SRH [22]. Furthermore, Spanish studies involving cohorts of women who were, on average, 10 to 20 years older than those in the present cohort reported lower SRH scores among women with sPOP [23, 24].

Although younger women of reproductive age were included in the abovementioned studies, age groups were not differentiated in the analyses, making it difficult to assess the specific impact of sPOP on SRH among this population group. Our study therefore contributes new knowledge by demonstrating that, 1 year after childbirth, women of reproductive age with sPOP had higher odds of reporting poorer SRH than those without sPOP.

Even though it is known that SRH reflects the multidimensional nature of health [14, 15], the question of what women with sPOP exactly convey in their responses to SRH when they rate it low remains open. Some people refer to specific health issues, while others consider their overall functioning when responding to the question [15]. The impairment of various facets of health is reflected, for instance, in quantitative Spanish research on women with sPOP without connection to recent childbirth, where vitality was identified as the most affected aspect of quality of life, followed by emotional and mental health components [24]. Qualitative studies from Australia [9], Ireland [7], and Sweden [8] further emphasize the severe impact of sPOP, including loss of sexual health, disturbances in urinary and bowel function, negative body image, reduced physical activity, fear of symptoms worsening, maternal insecurity, and psychological distress [7–9]—all of these indicate a broader decline in multiple dimensions of health and well-being. Partners of women with sPOP reported similar experiences, noting the women’s depression, difficulties bonding with their newborns, impaired intimacy, strained relationships, and social isolation [10].

Given SRH’s predictive value for future health [14, 15] it is concerning that 28.3% of new mothers with sPOP in our cohort rated their SRH as poor. Supporting this group benefits society, as these women have most of their active lives ahead, and maternal health is vital for parenting and women’s overall quality of life.

Not surprisingly, the covariates UI and FI were also associated with poor SRH, which is consistent with findings from both previous international and national research [24–27].

What distinguishes our study, is that we specifically examined isolated sPOP when adjusting for both FI and UI. We believe this distinction is important, as isolated sPOP can occur without concurrent pelvic floor conditions and, in our results account for 6% of women and may therefore require separate clinical attention.

The main strength of this study is the use of the national quality register with a large, unselected population and diverse variables. The uniqueness of this database lies in the use of PROM and PREM questions, where screening for sPOP is performed at a national level among Swedish women 1 year after childbirth. To our knowledge, no previous studies on sPOP have been conducted on such a large population of women of reproductive age. Although the response rate to both Surveys 1 and 3 was 28%, it still represents a substantial number of participants. Nevertheless, the large number of nonresponders could negatively influence the representativeness of the sample. According to the sensitivity analysis of nonresponders, a larger proportion of the women included in the study were first-time mothers, which can likely be explained by the fact that motherhood was a new experience for them, one they were more eager to share.

One limitation of this study is that the SRH item only labels the endpoints of the scale 1 (very poor) and 5 (very good), without descriptors for points 2, 3, and 4. This might have reduced the reliability of the scale. Furthermore, dichotomizing this variable may also be considered a limitation; however, this approach was chosen to ensure consistency with earlier research on similar populations [17, 18].

Further, variable sPOP was also dichotomized from five answer options (never and hardly ever into “no” and 1–3 times per month, 1–3 times per week or daily into “yes”) in the same way as in previous studies which used the same answer alternatives [2]. It was not a straightforward decision, given the difficulty of defining the boundary between health and disease from a medical perspective [21]. For instance, the study [28] on factors influencing POP surgery outcomes considered that patients had no sPOP (cured) if they reported experiencing vaginal bulging “never” or “hardly ever” or “1–3 times per month”. However, that study involved women with a mean age over 60, much older than in the current study. Older women may be more likely to accept symptoms as a normal part of aging, or positive surgical outcomes may be assessed more generously in this group.

The other Limitation of this study is the absence of baseline measurements verifying sPOP before pregnancy, especially for multipara. At the same time, adjusting for pre-pregnancy SRH strengthens the results. Given that SRH before pregnancy was the strongest predictor, associated with more than three times higher odds of poor SRH 1 year after childbirth, sPOP still showed a significant association. It may be beneficial to add PFD screening questions to the pre-pregnancy Survey 1, as this could help identify women with PFD and at risk of worsening symptoms after childbirth, allowing for earlier targeted interventions.

Our study was conducted 1 year after childbirth, when women are generally expected to have adapted to their new role as mothers and to bodily changes. While mild sPOP often improves during the first year [5, 29], it is likely that women in our cohort suffered more severe pelvic floor injuries, which may lead to a progression of symptoms requiring medical actions.

In conclusion, this nationwide study found that women with sPOP 1 year after childbirth rated their health lower than those without, highlighting sPOP as a public health issue in Sweden affecting women in their prime and potentially leading to long-term health problems. We support calls for comprehensive research on the medium- and long-term consequences following childbirth [1]. Future studies should target specific impairments caused by sPOP and modifiable risk factors. While progress is being made in diagnosis, treatment, and national maternity care initiatives [30], continued efforts are needed, especially in the prevention of sPOP and other PFDs, and, of course, in the ongoing development of effective treatments, surgical techniques, rehabilitation, support programs, and clinical guidelines aimed at improving outcomes for affected women.
